# Clinical evaluation of the efficacy of methylnaltrexone in resolving constipation induced by different opioid subtypes combined with laboratory analysis of immunomodulatory and antiangiogenic effects of methylnaltrexone

**DOI:** 10.1186/1472-684X-13-42

**Published:** 2014-08-20

**Authors:** Elisabeth CW Neefjes, Maurice JDL van der Vorst, Manon SA Boddaert, Wouter WA Zuurmond, Hans J van der Vliet, Aart Beeker, Hendrik P van den Berg, Cornelis J van Groeningen, Suzan Vrijaldenhoven, Henk MW Verheul

**Affiliations:** 1 Department of Medical Oncology, VU University Medical Center, Cancer Center Amsterdam, Amsterdam, The Netherlands; 2 Bardo Hospice, Hoofddorp, The Netherlands; 3 Department of Anesthesiology, VU University Medical Center, Amsterdam, The Netherlands; 4 Department of Internal Medicine, Spaarneziekenhuis, Hoofddorp, The Netherlands; 5 Department of Internal Medicine, Tergooi, Hilversum, The Netherlands; 6 Department of Internal Medicine, Amstelland Ziekenhuis, Amstelveen, The Netherlands; 7 Department of Internal Medicine, Medisch Centrum Alkmaar, Alkmaar, The Netherlands

**Keywords:** Constipation, Palliative care, Opioids, Methylnaltrexone, Immunomodulation, Angiogenesis

## Abstract

**Background:**

Opioid-induced constipation (OIC) is one of the major symptoms in palliative care with a prevalence of 30-50%. Methylnaltrexone for the treatment of OIC is significantly more effective than placebo, but only in about fifty percent of the patients regardless of dose increase. Dose increases cause increased toxicity without additional efficacy, and are therefore not recommended.

While methylnaltrexone is a μ-receptor antagonist, only a few opioids are solely μ-receptor agonists. Therefore, the response to methylnaltrexone may be determined by the receptor-profile of a specific opioid. In addition, methylnaltrexone may also affect the immune system and angiogenesis as was found in pre-clinical studies. Primary aim of this study is to determine differences in the efficacy of methylnaltrexone prescribed to resolve opioid induced constipation between three commonly used opioid subtypes: morphine sulphate, oxycodone and fentanyl. Secondary aim is to explore potential immunomodulatory and antiangiogenic effects of methylnaltrexone.

**Methods:**

In this multi-center, prospective, parallel group trial we will evaluate the efficacy of methylnaltrexone in resolving OIC occurring as a side effect of the most common opioid subtypes: morphine, oxycodone and fentanyl. In total 195 patients with OIC despite prophylactic laxatives will receive methylnaltrexone every other day up to fourteen days. Patients will report its effect in a laxation diary. Group allocation is based on the opioid type the patient is using. At the start and end of the study period patients complete the Bowel Function Index questionnaire. A subgroup of the patients will donate blood for analysis of immunomodulatory- and anti-angiogenic effects of methylnaltrexone.

**Discussion:**

In this study we aim to determine the efficacy of methylnaltrexone per opioid subtype to reduce constipation. We expect that the outcome of this study will improve the clinical use of methylnaltraxone.

**Trial registration:**

This trial is registered at clinicaltrials.gov: NCT01955213 and in the Dutch trial register: NTR4272.

## Background

Constipation is one of the major symptoms in palliative care, with a prevalence rate of 30-50% in patients with cancer [1,2]. It leads to a noticeable decrease in quality of life [3] by causing physical symptoms such as bloating and straining, and sometimes necessitates hospitalization [4]. Constipation can have multiple causes, which can be divided into anorectal dysfunction, or slow colon transit [5]. In palliative care for patients with cancer mechanical obstruction by tumor depositions or ascites might also be a cause of constipation, but often constipation is caused by opioid use. The incidence of opioid induced constipation (OIC) is estimated to vary between 35 and 70% of the patients using opioids [4,6-8]. This large range is probably caused by variation in type and dosage of opioids, the amount of (prophylactic) laxatives used and the way patients are monitored for this side effect in different studies [4,6-8]. The Rome criteria (currently version III) are generally used to diagnose constipation [5]. According to the Rome III criteria for constipation, a patient must have experienced at least 2 of the following symptoms over the preceding 3 months with symptom onset at least 6 months before diagnosis: fewer than 3 bowel movements per week, straining, lumpy or hard stools, sensation of anorectal obstruction, sensation of incomplete defecation, and/or manual maneuvering required to defecate. However, these diagnostic criteria might not be applicable to patients with OIC, because they require the presence of complaints for at least six months, while OIC usually develops within days to weeks and might need urgent treatment. Therefore, a practical definition of OIC is a decrease of the frequency of bowel movements after initiation of opioids to a frequency of less than three bowel movements per week [9]. To prevent OIC, guidelines recommend prophylactic laxatives [10,11]. Most patients are able to manage their bowel movements by in- or decreasing the dosage of the laxatives they use. However, 12-20% of the patients report symptoms of OIC despite laxative prescription [12]. For these patients methylnaltrexone, a peripherally acting μ-opioid receptor antagonist, can be a valuable therapeutic option.

Opioids exert their action through the opioid-receptors. These receptors are present in the central nervous system but also in peripheral tissues. Under normal conditions opioid receptors are receptive to endogenous opioids, such as enkephalins and endorphins [13]. The three major types of opioid receptors are the mu (μ), kappa (κ) and delta (δ) opioid receptor [13]. The affinity for the different receptors varies between different types of opioids [13]. How each receptor type contributes to OIC is not fully understood. There is evidence that each receptor type has an effect on gastro-intestinal motility and fluid secretion [14]. Previous studies suggest that these effects are mainly mediated by the peripheral opioid receptors [14-16].

### Methylnaltrexone

Methylnaltrexone (Relistor®) is an opioid receptor antagonist which blocks opioid binding at the μ-receptor but also has some affinity for the κ-receptor. It is a quaternary derivative of naltrexone with restricted ability to cross the blood–brain barrier. It therefore functions as a peripherally acting opioid antagonist, which reduces the opioid-induced decrease in gastrointestinal motility and delay in gastrointestinal transit time, and thereby reduces OIC. Methylnaltrexone does not affect opioid analgesic effects or induce opioid withdrawal symptoms. It is excreted by the kidneys and in the feces and has a plasma half life of approximately 8 hours. The most common side effects of methylnaltrexone are abdominal pain, nausea, flatulence, and diarrhea, which are likely to be related to increased peristaltic activity [17]. Methylnaltrexone has been approved by the FDA and EMA for patients who receive palliative care (regardless of medical condition) with OIC despite prophylactic laxative use.

Methylnaltrexone for the treatment of OIC is significantly more effective than placebo [9]. However, in both the randomized and open-label phases of the pivotal multi-center trial, methylnaltrexone produced rescue-free laxation in only about half the patients [9]. Dose increases did not influence these results [18]. There may be several reasons for this observation. First, constipation in palliative care patients may have multiple simultaneously occurring causes, unrelated to opioid therapy. Second, assuming that the constipation of the non-responders is still opioid-induced, one can hypothesize that the response to methylnaltrexone could be determined by the receptor-profile of the specific opioid the patient is using (Table 1) [4,6-8,19]. This is because methylnaltrexone is primarily a μ-receptor antagonist, while not all opioids are solely μ-receptor agonists. A frequently prescribed opioid like oxycodone is a κ-receptor and δ-receptor agonist [13] as well as μ-receptor agonist, whereas morphine and fentanyl are both mainly μ-receptor agonists. Morphine and fentanyl, on the other hand, differ in their lipid solubility and their tendency to sequestrate in the central nervous system [13]. This may possibly contribute to differences in response to methylnaltrexone, because methylnaltrexone is unable to pass the blood–brain barrier.

**Table 1:** Opioid characteristics regarding constipation

**Opioid**	**Receptor affinity**	**Lipophilicity**	**Distribution site**	**Incidence of constipation**^**1**^
**Morphine Sulphate**	Mainly μ, some κ	Low	Peripheral	60% (range 21-70%)
**Oxycodone**	μ, κ and δ	Intermediate	Central and peripheral	65% (range 25-74%)
**Fentanyl**	μ (highly selective)	High	Central	35% (range 10-55%)

^1^Estimated incidence of constipation without laxative use and the range of this incidence found in different studies in which a proportion of patients already uses laxatives.

In all previously mentioned studies no comparison was made between the response rates to methylnaltrexone of patients using different types of opioids [9,17]. Therefore, we recently initiated a clinical trial to determine whether different receptor-profiles of opioids are related to the efficacy of methylnaltrexone.

Besides constipation, there are other side effects of opioids that might be of clinical importance. Acute and chronic use of opioids is known to have inhibitory effects on humoral and cellular immune responses and may also have a stimulating effect on angiogenesis [20-24]. Μu-type opioid receptors are identified in several immunological cell subsets [25,26], and it has been demonstrated that opioids suppress T cell functioning and the production of several cytokines [23,27-29]. Activation of the μ-opioid receptor expressed on endothelial cells stimulates angiogenesis and a synergistic effect of morphine sulphate with VEGF has been demonstrated [21,30,31]. Methylnaltrexone could be of additive value to prevent this unwanted immunomodulatory and angiogenic activity of opioids [30]. Therefore, we will study the effects of methylnaltrexone on immunomodulatory and angiogenic activity in patients during treatment with this agent.

### Aims

Primary aim of the study is to compare the efficacy of a fixed dose of subcutaneous methylnaltrexone to induce laxation in patients who suffer from constipation due to either fentanyl, oxycodone or morphine sulphate despite optimal prophylactic laxative use.

Secondary aims are 1) to determine size, phenotype, and function of various leukocyte subsets as well as serum cytokine levels during treatment with the μ-opioid receptor antagonist methylnaltrexone and 2) to determine whether systemic antagonistic treatment with methylnaltrexone will modify systemic biomarkers of angiogenesis.

## Methods

**Figure 1:**
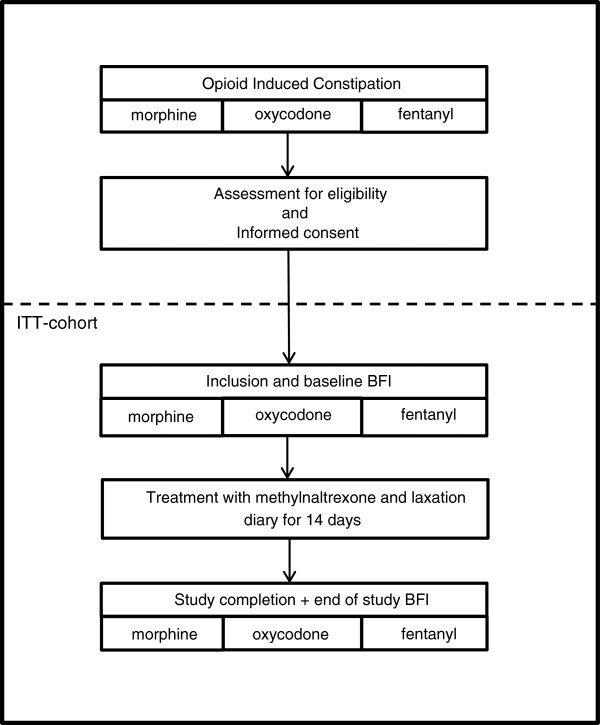
Study flow chart.

This study is a multi-center, prospective, parallel-group, observational study to compare the efficacy of methylnaltrexone between patient groups using different types of opioids. The immunomodulatory and anti-angiogenic effects of methylnaltrexone will be evaluated in a subset of patients included in the VU University medical center. The trial will be conducted in accordance with the Declaration of Helsinki and Good Clinical Practice guidelines and has been approved by the Medical research Ethics Committee of the VU University medical center and the institutional ethics committees of the other participating sites. Patient recruitment and data collection started in July 2012. A study flow chart is provided in Figure 1.

### Patients

Patients are recruited from the outpatient clinics and the inpatient wards of a University medical center, three teaching hospitals and a high care hospice in the Netherlands. We ask patients for informed consent to participate in this study if they suffer from OIC caused by morphine, oxycodone or fentanyl, despite prophylactic laxative use. Patients should use only one subtype of maintenance opioids, but (short-acting) rescue doses of another opioid subtype are allowed for up to two doses a day. Rescue doses of a short-acting form of the same opioid subtype as the maintenance opioid can be used in any dosing frequency. With this maximum of two doses the rescue opioid will not have a therapeutic level during a significant part of the day [13].

Patients should be on a stable laxative regimen for at least 3 days before the first dose of methylnaltrexone. This is defined as at least one type of laxative in an adequate dosing regimen, (e.g. macrogol 2 packets daily, magnesium(hydr)oxide 500 mg three times daily, bisacodyl 10 mg daily or sennoside A + B 10 ml daily) or at least two types of laxatives in a suboptimal dose with patient characteristics hampering optimal treatment.

Further inclusion criteria are that the patient should be aged ≥18 years; receives supportive care for any medical condition that requires prolonged opioid use (e.g. cancer or chronic obstructive pulmonary disease); is able to provide informed consent; has an opioid induced change in his/her laxation pattern with <3 bowel movements in the past week, and no bowel movement in the past 24 hours, or no bowel movement in the past 48 hours.

Exclusion criteria for participation are previous treatment with methylnaltrexone; a life expectancy of less than two weeks; presence of a fecal ostomy or an intraperitoneal catheter; clinically relevant active diverticular disease; body weight <38 kg; and contra-indications for methylnaltrexone use, such as a suspected mechanical gastrointestinal obstruction, impaired renal function (eGFR <30 ml/min/1.73 m^2^ or requiring dialysis), pregnancy or lactation, treatment with an investigational product, and presence of another, more probable cause of constipation. Presence of the latter is discussed between the patients physician and the investigator, and if necessary additional imaging or laboratory analysis is performed. Patients should be excluded if there are clinical abnormalities present that may interfere with participation or compliance to the study. Additional exclusion criterion for the laboratory part of the study is the use of immunomodulatory drugs, such as chemotherapy and tyrosine kinase inhibitors in the past four weeks, or the use of corticosteroids ≥ 30 mg prednisolone or equivalent in the past two weeks.

### Randomization, blinding and treatment allocation

Since all patients have an indication for treatment with methylnaltrexone and literature has already shown better responses to methylnaltrexone than to placebo, there will be no placebo treatment arm.

All patients receive the same dosing schedule of methylnaltrexone; there will be no randomization or blinding procedure. Study groups are defined by the type of opioid used by the patient, being either morphine sulphate, fentanyl or oxycodone.

### Treatment

Patients will be treated with methylnaltrexone in a standard dosing regimen for their weight:

38–62 kg: 8 mg

62–114 kg: 12 mg

>114 kg: 0.15 mg/kg

Methylnaltrexone will be administered subcutaneously every other day for up to 7 doses. Treatment with methylnaltrexone will be continued for up to 14 days. If desired, the patient may continue methylnaltrexone treatment after finishing the 14-day study treatment.

While enrolled in this study, it is not allowed to use rescue laxatives or enemas 4 hours before and/or after methylnaltrexone administration. Rescue laxatives are defined as laxatives which are not prescribed on a regular basis at the start of the study, and might be needed if methylnaltrexone is not effective. Standing laxative orders and opioid treatment will be continued during the study, but can be modified according to clinical judgment if diarrhea occurs or constipation persists. If needed, opioid dosing can be increased, however not decreased to a level below baseline. The use of spasmolytic drugs to relieve abdominal cramps is permitted in this trial.

### Study measurements

Demographic and baseline data of each patient will be extracted from his/her medical record and checked with the patient. These will include the medical condition for which the patient uses opioids, the daily dose of the specific opioid subtype the patient uses, and the type and dose of laxatives the patient uses.

Patients will be asked to complete a laxation diary from day -3 up to day 14, and the Bowel Function Index (BFI) on day 0 and 14. In the laxation diary the time of administration of methylnaltrexone is noted, as well as the timing, consistency and volume of bowel movements. Patients also note an average pain score for each day, the occurrence of side effects and use of rescue laxatives in the laxation diary. The BFI consists of three questions about symptoms of constipation experienced during the past week. Answers to these questions are rated on a scale from 0 to 100 and the final score is calculated by the mean of the three answers. A decrease of 12 points or more between start and end of the study is thought to be a clinically significant response [32].

From patients taking part in the exploratory study, blood will be drawn before the first administration of methylnaltrexone (day 0), after 24 hours (day 1), at day 14 and around day 42 for immuno- and angiogenic measurements.

### Non-responders

If the patient has no response to the first 4 administrations of methylnaltrexone (first week of treatment) the treatment will be halted. Patients should still complete the diary and BFI according to the study schedule.

### Adverse events

Adverse events are defined as any undesirable experience occurring to a patient during the study, whether or not considered related to the treatment. All adverse events reported spontaneously by the patient or observed by the investigator or his staff will be recorded and graded according to the common terminology criteria for adverse events version 4.0. All adverse events will be followed until they have abated, or until a stable situation has been reached. Depending on the event, follow up may require additional tests or medical procedures as indicated, and/or referral to the general physician or a medical specialist.

Diarrhea, abdominal cramps or sudden increase of pain grade 3 according to the Common Terminology Criteria for Adverse Events (CTCAE), that is related to the study drug according to the investigator, could be reason to skip the next dose of methylnaltrexone.

If these side-effects re-appear after the following dose, the treatment with methylnaltrexone should be halted. Patients will be asked to complete the laxation diary and BFI according to the study schedule. Patients participating in the laboratory part of the study will be withdrawn from this part of the study.

Diarrhea, abdominal cramps or sudden increase of pain grade 4 according to the CTCAE, that is related to the study drug according to the investigator, would be reason to stop treatment with methylnaltrexone. Patients will be asked to complete the laxation diary and BFI according to the study schedule, if possible. Patients participating in the laboratory part of the study will be withdrawn from this part of the study.

### Endpoints

Primary endpoint is the proportion of patients that has a rescue-free laxation response within 4 hours after at least 2 of the first 4 doses (the first week of treatment). Secondary endpoints are the time to first laxation, response percentages within 4 or 24 hours after the first and consecutive methylnaltrexone administrations, the number of laxations per week and the change in BFI score between day 0 and 14.

Additional study parameters are the change in leukocyte subsets and serum cytokine levels, angiogenic blood factor concentrations, level of endothelial progenitor cells, and angiogenic potential determined with in vitro endothelial cell proliferation assays.

### Sample size calculation and statistical analysis

Opioid treatment may cause constipation, but constipation may also have another etiology. In calculating the number of patients per group we assumed OIC in constipated patients treated with fentanyl to be 40% and in patients treated with morphine sulphate or oxycodone to be 70 and 75% [8]. Based on the receptor-profile and pharmacokinetics of the different opioids [13] we hypothesize that methylnaltrexone will induce successful laxation in 60% of the patients in the morphine group, 60% of the patients in the oxycodone group and in 25% of the patients in the fentanyl group within 4 hours after at least 2 of the first 4 doses (Table 2). For testing two hypotheses, being a 35% difference in response rate between the morphine group vs. the fentanyl group and a 35% difference in response rate between the oxycodone group vs. the fentanyl group, we used a corrected α of .025 instead of .05. This hypotheses will be tested against the null-hypothesis that there is no difference between the efficacy of methylnaltrexone between the different opioid subtypes. Calculation of the sample size performed with STATA 11, using a power of 0.8 and an α of .025 in a two sided test, results in 62 patients in the morphine sulphate group, 62 patients in the oxycodone group and 31 patients in the fentanyl group. Taking into account an expected drop-out rate of 20% we aim to include 78, 78 and 39 patients respectively.

**Table 2:** Expected response rate to methylnaltrexone

**Opioid**	**Probability that constipation is opioid induced**	**Occupation of the peripheral μ receptor by opioid subtype**	**Expected response rate to methylnaltrexone**
**Morphine Sulphate**	70%	High	60%
**Oxycodone**	75%	Intermediate-high	60%
**Fentanyl**	40%	Low	25%

Based on the data in Table 1, we expect a high probability that the constipation is opioid induced for morphine sulphate and oxycodone. This is in contrast with a low probability that it is opioid induced for fentanyl. Because morphine sulphate primarily acts on the peripheral mu receptor, we expect a good response rate to methylnaltrexone. The combination of a high probability that the constipation is opioid induced in the oxycodone group, with its activity on both the central and the peripheral mu, kappa, and gamma receptor leads to an expected response rate that equals that of morphine sulphate. For the fentanyl group both the low probability that the constipation is opioid induced, and the sequestration in the central nerve system lead to a very low expected response rate to methylnaltrexone.

Based on the sample size of the three groups it seems feasible to include 20 patients of each group in the exploratory part of the study, in which the immunologic and angiogenic effects of methylnaltrexone are studied. This sample size seems to be large enough to account for the inter individual differences in the results found in previous studies on this subject.

Data will be anonymized and collected in an web-based database system (Open Clinica). Statistical analysis will be performed in SPSS version 20. The primary endpoint will be expressed in the proportion of patients having rescue-free laxation response within 4 hours after at least 2 of the first 4 doses and significance will be evaluated by the *χ*^2^ test.

The proportion of patients fulfilling the secondary end points will also be evaluated by the *χ*^2^ test. The time to laxation, the number of laxations per week, the change in BFI score and the data from the laboratory part will be continuous variables and will therefore be presented by their mean and standard deviation and analyzed by means of the student *t*-test, Mann–Whitney *U* test, or ANOVA, whichever is considered most appropriate.

## Discussion

Constipation is one of the most common symptoms in palliative care, and is frequently caused by opioids. Despite prophylactic laxatives, up to 20 percent of patients using opioids will develop opioid induced constipation (OIC). Methylnaltrexone, a peripherally acting μ-opioid receptor antagonist, is designed to displace the opioid from peripheral receptors in the gut, thereby decreasing the opioid's constipating effects and inducing laxation, without reducing analgesia.

In current practice it is used as rescue medication after failure of standard (combination) laxative therapy. Methylnaltrexone prescribed in an earlier stage could avoid severe OIC symptoms. The reasons for this rescue strategy are mainly based on the fact that methylnaltrexone has only been tested as rescue medication for OIC and that the costs of methylnaltrexone are higher than those of other laxatives. Reports of gastrointestinal perforations after use of methylnaltrexone might have resulted in more reluctance to prescribe this drug [33]. Another important factor that tempers the use of methylnaltrexone is the observation that only half of the patients respond to this treatment. We hypothesize that the response rate is dependent on the receptor profile of the opioid that is causing constipation. Consequently, it should be possible to optimize clinical benefit of methylnaltrexone by prescribing this in an earlier stage of constipation treatment to patients who are likely to respond. In this study we will evaluate differences in efficacy of methylnaltrexone in reducing OIC between the commonly prescribed opioid subtypes morphine sulphate, oxycodone and fentanyl.

As this is a prospective study, we have the benefit of adequate power compared to a subset analysis of previous studies. We will evaluate the objective response (number of laxations) in combination with the clinical benefit as is rated on the Bowel Function Index.

In this study the opioid subtypes the patients are using are not randomized, but based on the preference of the treating physician and the side effects experienced by patients. Although this might influence the incidence of OIC, it should not influence the efficacy of methylnaltrexone for a specific opioid subtype.

We have not included a placebo treatment group, because methylnaltrexone is already proven effective and cannot be withheld from patients for ethical reasons.

In this study rescue opioids of a different subtype than the maintenance opioid are allowed, with a maximum of two rescue doses per day. This decision is based on the fact that in daily practice a majority of patients use opioid combination regimens with a difference in prescribed subtypes of rescue- and maintenance opioids. We have set a cut-off at two rescue-opioid doses a day. This is based on the pharmacokinetic properties of rescue opioids, which will not have therapeutic levels during a significant part of the day when they are administered twice a day [13]. The possibility that these rescue opioids contribute to the development of OIC, even in these low doses, can however not be ruled out.

The exploratory laboratory part of this study is of particular interest when the anti-angiogenic and immunomodulatory effects found in pre-clinical studies are clinically confirmed. If methylnaltrexone inhibits this pro-angiogenic and immunomodulatory effects of opioids, a randomized controlled trial investigating the effects of opioids with or without methylnaltrexone on tumor progression and survival could be a next step.

## Competing interests

The authors declare that they have no competing interests.

## Authors’ contributions

ECN participated in the design of the study, coordinates the conduct of the trial and drafted the manuscript. MJV contributed to the set up of the study and helped to draft the manuscript. MSB was involved in the design of the study protocol and contributes to the conduct of the trial. WWZ contributed to the design of the study. HJV contributed to the design of the study. AB contributes to the conduct of the trial. HPB contributes to the conduct of the trial. CJG contributes to the conduct of the trial. SV contributes to the conduct of the trial. HMV was involved in the design of the trial and helped to draft the manuscript. All authors read and approved the final manuscript.

## Pre-publication history

The pre-publication history for this paper can be accessed here:

http://www.biomedcentral.com/1472-684X/13/42/prepub
